# Improvement of Insight with Non-Invasive Brain Stimulation in Patients with Schizophrenia: A Systematic Review

**DOI:** 10.3390/jcm11010040

**Published:** 2021-12-22

**Authors:** Martin Blay, Ondine Adam, Rémy Bation, Filipe Galvao, Jérôme Brunelin, Marine Mondino

**Affiliations:** 1 Centre Hospitalier le Vinatier, F-69500 Bron, France; martin.blay5@gmail.com (M.B.); ondine.adam@ch-le-vinatier.fr (O.A.); filipe.galvao@ch-le-vinatier.fr (F.G.); jerome.brunelin@ch-le-vinatier.fr (J.B.); 2 Université Lyon 1, Lyon University, F-69100 Villeurbanne, France; remy.bation@chu-lyon.fr; 3 INSERM U1028, CNRS UMR5292, PSYR2 Team, Lyon Neuroscience Research Center, F-69000 Lyon, France; 4 Psychiatric Unit, Wertheimer Neurologic Hospital, F-69500 Bron, France

**Keywords:** illness awareness, neuromodulation, electroconvulsive therapy, transcranial magnetic stimulation, transcranial electrical stimulation, transcranial direct current stimulation, transcranial alternating current stimulation, transcranial random-noise stimulation

## Abstract

Patients with schizophrenia are often unaware of their condition and the consequences of their illness. This lack of insight results in impaired functioning, treatment non-adherence and poor prognosis. Here, we aimed to investigate the effects of non-invasive brain stimulation (NIBS) on two forms of insight, clinical and cognitive, in patients with schizophrenia. We conducted a systematic review of the literature registered in the PROSPERO database (CRD42020220323) according to PRISMA guidelines. The literature search was conducted in Medline and Web of Science databases based on studies published up until October 2020 that included pre-NIBS and post-NIBS measurements of clinical and/or cognitive insight in adults with schizophrenia. A total of 14 studies were finally included, and their methodological quality was assessed by using the QualSyst tool. Despite the lack of well-conducted large randomized-controlled studies using insight as the primary outcome, the available findings provide preliminary evidence that NIBS can improve clinical insight in patients with schizophrenia, with a majority of studies using transcranial direct current stimulation with a left frontotemporal montage. Further studies should investigate the effect of NIBS on insight as a primary outcome and how these effects on insight could translate into clinical and functional benefits in patients with schizophrenia.

## 1. Introduction

Schizophrenia is a severe psychiatric disorder affecting 20 million people worldwide [1] and is associated with poor functional, social and professional outcomes, and with increased early mortality [2]. Schizophrenia is characterized by a combination of positive symptoms, including delusions and hallucinations; negative symptoms such as apragmatism and social withdrawal; and/or disorganization symptoms [3]. One of the core features of schizophrenia is the lack of insight into the illness, i.e., the lack of awareness of having a mental disorder, of its symptoms and of its consequences, which is observed in 50% of patients with schizophrenia [4,5]. This feature has been shown to be linked to several cognitive functions, including the theory of mind [6]. The lack of insight largely contributes to impaired functioning, treatment non-adherence, poor outcomes and poor prognosis [7,8].

In recent years, two main forms of insight, clinical and cognitive, have been distinguished and appear to involve different brain networks [9]. *Clinical insight*, which was initially considered as a dichotomous construct that was either present or absent, is now recognized as a multidimensional construct that exists on a continuum consisting of domains including awareness of having a serious mental illness, awareness and attribution of symptoms to the illness; acceptance of the need for treatment; and awareness of social, occupational or other negative consequences of the disorder [10]. Clinical insight is thought to be related to multiple brain structures including prefrontal and temporoparietal regions, with an inter-hemispheric imbalance where the left hemisphere is hyperactive when clinical insight is lacking [11,12]. *Cognitive insight* refers to the ability of patients to evaluate their anomalous self-experiences and to correct distorted beliefs and misinterpretations. Cognitive insight involves two subcomponents: the capacity for self-reflectiveness and the capacity to resist excessive self-certainty [13,14,15]. This form of insight is thought to be related to specific brain regions: the hippocampal neural efferent pathway being involved in self-certainty [16] and the prefrontal (especially its ventrolateral part) and temporal cortical regions being involved in self-reflectiveness [17]. Although they are thought to be different constructs, the different forms of insight are intimately correlated, and cognitive insight is sometimes seen as prerequisite for clinical insight [13,18].

Usual pharmacological treatments are partly effective on insight. In a review examining results from 14 randomized controlled trials, Mattila et al. found that second-generation antipsychotic medications were associated with improvements in insight in patients with schizophrenia [19]. However, numerous patients still had impairments in insight into the illness even during treatment with second-generation antipsychotic. Furthermore, some psychological interventions have been developed to improve insight impairment in schizophrenia. In this line, cognitive-behavioural therapy for psychosis has been linked with improvements in clinical and cognitive insight [20,21,22]. Psychoeducation programs have also been developed, but it has been reported that while they can improve knowledge about the illness, this improvement did not always translate into improvement of insight [23]. More recently, new methods called metacognitive interventions were found to be effective in improving clinical and cognitive insight [24,25]. These metacognitive interventions encompass cognitive-behavioural therapy for psychosis, metacognitive therapy, metacognitive reflection insight therapy and metacognitive training, with more evidence of efficacy for the latter technique. However, such psychological interventions are not easily available, which limits their dissemination in clinical settings. Moreover, since patients with lack of insight are more likely to refuse to take and/or to stop taking antipsychotic medications [26,27] and to participate to psychoeducational or cognitive programs, it is of importance to develop new, low-cost and easily accessible treatments targeting insight impairment [7].

Non-invasive brain stimulation (NIBS) are emerging tools that allow modulation of brain activity and connectivity by applying electrical or magnetic stimulation over the scalp of a participant [28]. The oldest method of NIBS proposed as alternative strategy to alleviate symptoms in patients with neuropsychiatric condition is electroconvulsive therapy (ECT). Since the 1990s, alternative methods such as repetitive transcranial magnetic stimulation (rTMS) and transcranial direct current stimulation (tDCS) have been suggested for alleviating symptoms and improving cognition in patients with neuropsychiatric condition [29]. In patients with schizophrenia, rTMS and tDCS have shown promising results to reduce medication-resistant auditory verbal hallucinations [30,31,32], negative symptoms [33] and to improve cognitive functioning [34]. However, some studies failed to demonstrate any superiority of active rTMS or active tDCS over sham to decrease symptoms of schizophrenia, e.g., [35,36] suggesting a high response variability to NIBS and limiting their clinical applicability. The rationale for using NIBS in schizophrenia was that stimulating brain regions related to schizophrenia symptoms would restore their normal functioning and, therefore, reduce symptoms. In most cases, NIBSs were applied on prefrontal and temporoparietal areas. As these brain regions are also involved in insight, one may hypothesize that NIBS may also have beneficial impacts on insight.

Here, we conducted a systematic review of the available literature on the effects of NIBS on clinical and cognitive insight in patients with schizophrenia. The aim of the current review was to provide a current state of the art on the interest of using NIBS for improving insight in schizophrenia. We separately examined the effect of the different methods of NIBS on the different forms of insight in order to enlighten whether one method produced greater effects or whether one form of insight was more responsive to NIBS. Since different but partly overlapping brain networks are suggested to underlie each dimension of insight, we hypothesized that NIBS targeting distinct cortical regions may differentially impact each dimension. We also investigated whether NIBS-induced changes in insight were associated with changes in clinical symptoms.

## 2. Materials and Methods

Our systematic review was performed according to the recommendations from the Preferred Reporting Items for Systematic Reviews and Meta-Analyses guidelines (PRISMA, [37]). The protocol for this review was prospectively registered in the International Prospective Register of Systematic Reviews on 12 December 2020 (PROSPERO, registration number: CRD42020220323, which is available at the following website: https://www.crd.york.ac.uk/prospero/display_record.php?ID=CRD42020220323, accessed on 15 November 2021).

### 2.1. Literature Search Strategy

We searched for articles published up until October 2020 in MEDLINE and Web Of Science databases using the following combinations of keywords: (“Schizophrenia” OR “hallucinat*” OR “negative symptoms”) AND (“insight” OR “awareness” OR “metacogniti*”) AND (“rTMS” OR “repetitive transcranial magnetic stimulation” OR “transcranial magnetic stimulation” OR “TBS” OR “theta burst stimulation” OR “transcranial direct current stimulation” OR “tDCS” OR “HD-tDCS” OR “tACS” OR “transcranial alternative current stimulation” OR “tRNS” OR “transcranial random noise stimulation” OR “tES” OR “transcranial electrical stimulation” OR “ECT” OR “electroconvulsive therapy”). The search equation syntax was adapted for each database. We also searched for additional articles in reference lists of retrieved articles. 

### 2.2. Eligibility Criteria and Study Selection

We included every available original study (including randomized controlled trials (RCTs), cohort studies, case-control studies, cross-sectional studies, case reports and case series) published in peer-reviewed journals that investigated the use of NIBS in adults with schizophrenia or schizo-affective disorder, diagnosed using any recognised diagnostic criteria (Diagnostic and Statistical Manual of Mental Disorders—DSM; International Classification of Diseases—ICD), with an assessment of clinical and/or cognitive insight as primary or secondary outcome. We excluded meta-analyses, reviews, commentaries, editorials, conference abstracts, and book chapters. Animal studies and studies published in other languages than English were also excluded. The included studies had to provide at least one session of NIBS including ECT, rTMS, intermittent or continuous theta burst stimulation (TBS), tDCS, high-definition tDCS (HD-tDCS), transcranial alternative current stimulation (tACS) and transcranial random noise stimulation (tRNS). The included studies had to assess insight by means of any standardized psychometric scales.

Two investigators (M.B. and O.A.) independently screened all studies that resulted from the search and applied eligibility criteria to select studies for inclusion in the systematic review. Disagreements between individual judgements were resolved by a third investigator (M.M.), with pre-defined inclusion criteria.

### 2.3. Data Extraction and Quality Assessment

The following information was extracted from each individual study: study design, sample size, sociodemographic and clinical characteristics; NIBS technique and parameters; psychometric scales used to measure insight; and summary of results. The methodological quality of each included study was assessed with a standardized quality assessment criteria tool: the 14-item “QualSyst” tool [38]. A summary quality score was calculated for each study by summing the total score obtained across relevant items and dividing by the total possible score. Items specific to RCTs and not applicable to case-reports and open-label studies (e.g., randomization, blinding) were marked as “not applicable” (NA) and were excluded from the calculation of the summary score.

## 3. Results

### 3.1. Selection and Description of the Studies

**Figure 1:**
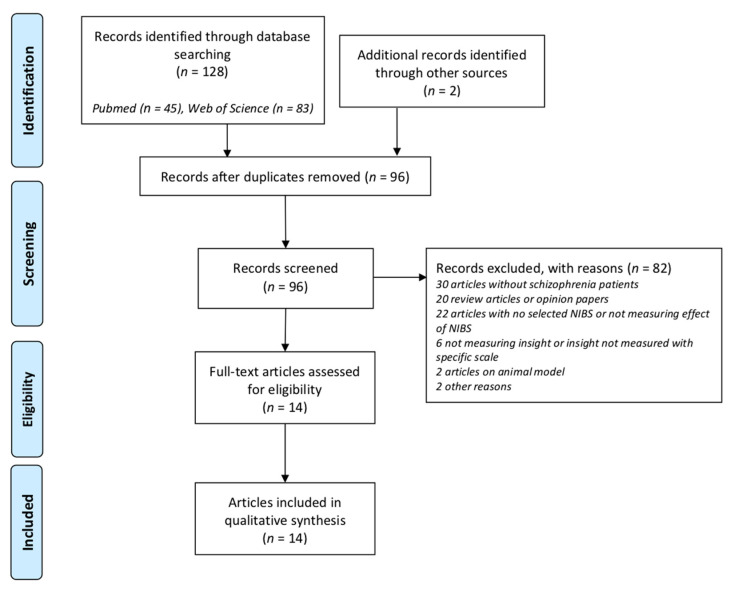
PRISMA 2009 flow diagram describing the selection procedure of the studies investigating the effects of non-invasive brain stimulation (NIBS) on insight in patients with schizophrenia. PRISMA indicates Preferred Reporting Items for Systematic Reviews and Meta-Analyses.

A total of 130 articles were identified through the literature search. After the removal of duplicates, 96 papers were screened for eligibility. Fourteen articles met our selection criteria and were, therefore, included in the systematic review. The literature search following PRISMA guidelines is depicted in Figure 1.

We selected six RCTs [39,40,41,42,43,44], three open-label studies [45,46,47], one case series [48] and four case reports [49,50,51,52]. The studies came from five independent research groups. Three out of six RCTs included the same sample of patients but examined different clinical and neuropsychological outcomes [40,41,43]. Two out of three open-label studies included at least partially overlapping samples of patients [45,46]. Details and data extracted from each selected study are reported in Table 1.

Most of the selected articles used tDCS (nine studies). Only one study was found for each of the other NIBS techniques (tACS, tRNS, rTMS, ECT and HD-tDCS). We reported the effects of each technique independently. The large majority of studies used clinical assessment tools as primary outcome but two reported using a score of insight as their primary outcome [40,46].

### 3.2. Characteristics of the Patients Included in the Selected Studies

In total, the included studies recruited 226 patients, with mean age ranging from 24 to 46.4 and a sex-ratio of 1.073 (117 males and 109 females). Almost all the patients were right-handed: Nine studies included exclusively right-handed patients [40,41,43,44,45,48,50,51,52]; two studies reported to have included mixed samples with non-right-handed patients [39,47]; three studies did not provide information regarding handedness; and Kim et al. (2019) reported a mean score of 77.6 ± 16.2 at the Edinburgh Handedness Inventory [53]. Most of the patients included were on antipsychotic medication but two case-reports included drug-free patients [50,51]. The six RCTs included a mixed sample of patients with schizophrenia and schizoaffective disorders [39,40,41,42,43,44]. The other articles exclusively included patients with schizophrenia. Almost half of the studies included patients with treatment-resistant auditory hallucinations (6 out of 14) [40,41,43,45,46,47] or negative symptoms (3 out of 14) [39,42,48]. One study specifically included patients with impaired illness awareness [44]. The four remaining case reports included difficult-to-treat patients.

### 3.3. Means of Assessment of Insight

Overall, clinical insight was the most studied dimension of insight. Six different clinician-rated and self-rated scales were used to measure clinical insight: the Scale for Assessment of Unawareness of Mental Disorder (SUMD) was used by three studies [48,49,50] in its original version [54] and by two studies [39,41] in its abbreviated version [55], the Birchwood Insight Scale (BIS, [56]) was used by three studies [42,51,52], the Schedule for Assessment of Insight (SAI [10]) was used by two studies [45,46], the Self-Appraisal of Illness Questionnaire (SAIQ [57]) by two studies [39,43] and the VAGUS insight into psychosis scale was used by two studies [44,47], either with the version rated by the clinician (VAGUS-CR: clinician-rated) or the one rated by the patient (VAGUS-SR: self-reported) [58]. Finally, two studies [40,43] used the G12 “lack of judgement and insight” single-item score of the positive and negative syndrome scale (PANSS) [59] (range 1–7 with higher score representing lower insight) to provide a measure of clinical insight, associated with another scale measuring clinical or cognitive insight.

Cognitive insight was studied only with the Beck Cognitive Insight Scale (BCIS) [13], individually in one study and jointly with a standardized psychometric scale assessing clinical insight in two other studies. When used, BCIS was subdivided into a BCIS-R subscore, which reflects reflective attitude (self-reflectiveness), and a BCIS-C subscore, which reflects certain attitude (self-certainty), and a R-C (reflective attitude minus certain attitude) index was computed, with lower R-C index scores indicating poorer cognitive insight.

Here, we studied the effect of each NIBS on each dimension of insight independently to investigate whether one dimension was more responsive to each treatment.

### 3.4. Studies Investigating the Effect of tDCS on Insight

#### 3.4.1. Effects of tDCS on Clinical Insight

Five RCTs, two open-label studies and two case-reports assessed the effect of tDCS on clinical insight (Table 1). All of them delivered active tDCS with an intensity set at 2 mA during 20 min. The tDCS regimen consisted of 10 sessions of tDCS delivered twice a day (separated by at least 2 to 3 h) on five consecutive days in all studies except the one from Kim et al. (2019), which assessed the effects of a single tDCS session [44].

In three of the five RCTs [40,41,43], which are based on the same sample of patients but with different measures of clinical insight, the anode was placed over the left dorsolateral prefrontal cortex (between F3 and FP1 according to 10/20 international EEG system) and the cathode over the left temporoparietal junction (between T3 and P3). The authors found the following: (i) a significant improvement at the SUMD “awareness of disease” and “awareness of positive symptoms” dimensions with a moderate effect size at day 5, which persisted at 1 month but not at 3 months follow-up [41]; (ii) a significant increase at the SAIQ “need for treatment” and “presence/outcome of illness” subscales at day 5, which reduced to trend-level at 1 month follow-up [43]; and (iii) a significant decrease at the PANSS-G12 single item score [43]. Of note, the results on insight measured by the PANSS-G12 single item failed to reach statistical significance when corrected for multiple comparison and adjusted with illness duration and baseline depression severity scores [40,43]. Interestingly, an improvement of clinical insight was observed following frontotemporal tDCS with the same parameters in two case reports [51,52] and two open-label studies [45,46], which additionally reported a positive correlation between improvement in insight and improvement in auditory hallucinations.

The two remaining RCTs used different electrode placement. Namely, Kim et al. (2019) tested two different tDCS montages: a biparietal montage with the anode and cathode placed over P4 and P3, respectively, and a bifrontal montage with the anode and cathode placed over F4 and F3, respectively [44]. They found no significant effect of one session of either biparietal or bifrontal tDCS on clinical insight measured by the clinician-rated and self-reported VAGUS scales. In an RCT assessing the effects of frontal tDCS on negative symptoms as the primary outcome, Chang et al. (2021) used an electrode montage with two anodes placed over the points midway between F3 and Fp1 and between F4 and Fp2, respectively, and two extracephalic cathodes placed as references over bilateral forearms [39]. They reported a significant improvement of clinical insight at day 5, as measured by the SUMD “awareness of positive symptoms” (still significant at 3 months), “awareness of disease” (still significant at 1 month but not at 3 months) and “awareness of negative symptoms” (not significant at 1 month) dimensions. However, no significant effects were found on the self-reported measure of insight, as assessed by SAIQ.

Finally, an open-label study designed to investigate the usefulness of HD-tDCS on persistent auditory hallucinations in schizophrenia also reported a significant increase in clinical insight as measured using the VAGUS-CR scale after HD-tDCS sessions [47]. HD-tDCS was delivered at 2 mA for 20 min, twice a day, for 5 days using a 4 × 1 ring montage to target the left temporoparietal junction with the cathode placed at CP5 as the central electrode, surrounded by the four return electrodes (placed at FC3, FT7, PO7 and P1). The improvement in insight observed after HD-tDCS was positively correlated with the improvement in depressive symptoms assessed by the Montgomery–Åsberg Depression Rating Scale (MADRS).

#### 3.4.2. Effects of tDCS on Cognitive Insight

Three RCTs investigated the effects of tDCS on cognitive insight. Chang et al. (2019) reported a significant effect of frontotemporal stimulation on the “self-reflectiveness” dimension of the BCIS (moderate effect size) and on the R-C index (mild effect size) but not on the “self-certainty” dimension of the BCIS after the 5 days of tDCS (10 sessions), without persistence of the effect at one month [40]. The change in cognitive insight was positively correlated with the change in planning abilities measured by the Tower of London test. By contrast, no significant effects were reported on cognitive insight after either one session [44] or 10 sessions [39] of active bifrontal tDCS as compared to sham.

### 3.5. Studies Investigating the Effect of Other NIBS on Clinical Insight

Only one RCT investigated the effects of rTMS on clinical insight in patients with schizophrenia [42]. rTMS was delivered twice a day for 3 weeks (30 sessions) at 10 Hz: the first session of each day targeted the left DLPFC (the coil was placed over F3) and the second targeted the right DLPFC (the coil was placed over F4) with a 75-millimetre figure-of-eight coil. A significant improvement in insight as measured by the BIS score was reported after active rTMS treatment as compared to sham. The beneficial effect lasted up to 3 months. The effect on insight was mainly driven by an increase in the “awareness of the need for treatment” dimension.

One case-series and two case reports reported significant improvement in clinical insight in patients with schizophrenia treated with other NIBS techniques, namely after 20 sessions of bifrontal transcranial alternating current—tACS delivered at 4.5 Hz in three schizophrenia patients under a clozapine treatment [48]; after 10 sessions of frontotemporal transcranial random-noise stimulation—tRNS delivered at high frequency (between 100 and 640 Hz) in a drug-free patient with schizophrenia [50]; and after bilateral ECT delivered three times a week (pulsewidth, 1.0; frequency, 60 Hz; duration, 6.0 s; charge, 576 mC; propofol, 80 mg; succinylcholine, 40 mg; seizure duration, 27–59 s) in a patient with refractory schizophrenia with a strong affective component [49].

### 3.6. Quality Assessment

The results of the quality assessment with the standardized quality assessment criteria tool [38] are presented in Table 2.

Individual quality scores of studies ranged from 0.75 to 0.93 (average quality score of 0.89, SD = 0.07) for RCTs and from 0.58 to 0.85 (average quality score of 0.76, SD = 0.08) for open-label studies and case reports. The items with the lowest ratings are related to the sample size (criteria 9) for RCTs and to the study design (criteria 2) for case reports and open-label studies.

## 4. Discussion

The current systematic review provided an overview of all the available literature on the effects of NIBS on clinical and cognitive insight in patients with schizophrenia. In addition to the low number of available studies and the lack of well-designed RCTs with clinical and/or cognitive insight as primary outcome, we believe that this review enlightens interesting questions about this emerging field and opens new perspectives for the use of NIBS to improve insight in schizophrenia.

### 4.1. NIBS Effects on a Specific Form of Insight?

In the current review, we explored whether NIBS might improve one specific form of insight, i.e., clinical or cognitive, in patients with schizophrenia. Almost all selected studies found a significant increase in clinical insight following repeated sessions of NIBS in this population, with some studies even reporting a maintenance of the effect for at least 3 months. These results should nevertheless be taken with caution as they come from five RCTs but only from three independent samples of patients. In the current state of knowledge, most evidence came from studies using left frontotemporal tDCS, with the anode placed over the left prefrontal cortex and the cathode over the left temporoparietal junction (parameters: 20 min sessions conducted twice a day for 5 days, resulting in a total number of 10 sessions). Remarkably, all following subdimensions of clinical insight were reported as improved by NIBS: awareness of disease [39,41,49,50,52], awareness of positive symptoms [39,41,49,50], awareness of negative symptoms [39] and awareness of need for treatment [42,51,52]. These findings support a global effect on clinical insight rather than an effect on a specific subdimension.

Conversely, results remain unclear regarding cognitive insight. Some studies reported a beneficial effect while some others failed to observe a significant effect of repeated or single sessions of NIBS [39,40,44]. On the one hand, left frontotemporal stimulation with tDCS was associated with a significant improvement on self-reflectiveness but not on self-certainty dimension of cognitive insight [40], and on the other hand bifrontal stimulation with tDCS did not result in any cognitive insight improvement [39,44]. The current review did not provide us with sufficient data to conclude the effects of NIBS on cognitive insight.

### 4.2. NIBS-Induced Improvement in Clinical Insight: Cause or Consequence of Symptoms Improvement?

The mechanisms by which NIBS may induce beneficial effects on clinical insight remain unclear. One could infer that the insight improvement is mediated by—or at least linked with—the improvement in other symptoms of schizophrenia. The study of the link between insight and symptomatology has a long history in schizophrenia. A rich body of evidence has associated poor insight to increased overall, positive and negative symptom severity [60,61]. However, some longitudinal studies showed no clear relationship between changes in insight and improvements in symptoms [62]. Regarding NIBS literature, four studies investigated the statistical correlation between improvement in insight and improvement in targeted symptoms of schizophrenia. One RCT found that NIBS-induced insight improvement was significantly correlated to the improvement in overall schizophrenia symptomatology [39], and two open label studies found this improvement to be correlated to the improvement in auditory hallucinations [45,46]. Conversely, one RCT has reported a beneficial effect of tDCS on insight but not on other symptoms of schizophrenia, suggesting that these effects can occur independently of each other or at least not be concomitant [41]. Whether NIBS effects on insight are the cause or the consequences of NIBS effects on symptomatology is, thus, an open question that deserves further investigations.

### 4.3. NIBS-Induced Insight Improvement: A Pro-Cognitive Effect of NIBS?

In addition to the beneficial effect of NIBS on clinical symptoms observed in patients with psychiatric conditions, numerous studies have reported that NIBS can also improve cognition [29]. These pro-cognitive effects of NIBS have been observed in both healthy volunteers [63] and patients with psychiatric conditions [64], although evidence is sparse in patients with schizophrenia [65]. In addition, cognitive effects have been reported for both basic cognitive processes (including executive function, planning, attention and memory); social cognition [66,67] including theory of mind [68]; and metacognitive self-evaluation [69]. Given that cognitive insight has been associated with cognitive functioning such as premorbid IQ, executive functions (for a review see [7]), reality processing and declarative memory [70], one can hypothesize that NIBS-induced aftereffects on cognitive functioning may also translate into cognitive insight improvement in patients. In line with this, Chang et al. (2019) reported a significant positive correlation between tDCS-induced improvements of cognitive insight (R-C index scores at the BCIS) and planning abilities (measured by the accuracy at the Tower of London test). The relationship between cognition, social cognition, metacognition and insight abilities in patients with schizophrenia needs further investigation.

### 4.4. Insight Improvement: Translation into Better Functional Outcomes?

The potential functional consequences of an improvement of insight have not been systematically addressed in the reviewed studies. Two studies reported an improvement of insight and medication adherence following tDCS [39,43]. However, these improvements in insight and medication adherence were not significantly correlated [39,43].

There is a growing body of evidence that, if better clinical insight increases medication adherence, therapeutic alliance, community functioning and lessens intensity of symptoms, it is also associated with depression, poorer self-reported quality of life and suicide [7]. Likewise, if better cognitive insight is associated with lower levels of overall psychopathology, it is also associated with depression and heightened suicidality [7]. This association between higher levels of insight and increased levels of depression is called the “insight paradox” and is one of the major challenges in NIBS trials aiming at improving insight of patients with schizophrenia [71]. In the articles studied in this review, Sreeraj et al. (2018) found a correlation between insight improvement and the improvement in MADRS score, which is not consistent with the expected worsening depressive symptoms associated with improved insight. Only Kao et al. (2020) studied impacts on quality of life. They did not find any worsening on this outcome, with even an improvement on the psychological domain immediately after tDCS [43].

Further studies need to focus on these outcomes in order to have a holistic vision on the impact of these therapeutics on the patients and in order to provide a better evaluation of the benefit/risk balance of using NIBS as new therapeutic options. Careful attention should be paid on the evaluation of the potential consequences of insight improvement (especially on depressive symptoms, suicidal thoughts and quality of life).

### 4.5. NIBS Effects on Clinical Insight: A Neurobiological Explanation?

Our review identified that the majority of studies using frontotemporal tDCS reported a beneficial effect of stimulation on clinical insight in patients with schizophrenia. In this electrode montage, the anode was placed over the left prefrontal cortex and the cathode over the left temporoparietal junction. Although this electrode montage was initially developed to alleviate auditory hallucinations based on neuroimaging findings [30], it seems consistent to observe that stimulating the left frontotemporal network may also increase insight. Indeed, even if lack of insight is a complex phenomenon with poorly understood neurobiological substrate, several brain regions including superficial and deep structures, as well as anterior and posterior structures, have been implicated, and the left frontotemporal network is one among these [12]. Supporting this perspective, targeting either the DLPFC with bifrontal stimulation with tDCS [39], tACS [48] and rTMS [42] or the left temporoparietal junction by HD-tDCS stimulation [47] may help to increase clinical insight. One could, thus, infer that the effect on clinical insight may be supported by an effect of repeated sessions of NIBS on a part of the dysfunctional network of insight, either the left prefrontal cortex or the left temporoparietal junction or the connectivity between both. However, since the left frontotemporal region is the most targeted region in tDCS studies, the possibility that this region does not have any specificity vis-à-vis other brain areas to improve insight cannot be excluded. Further studies are needed to disentangle the involvement of the prefrontal cortex, the temporoparietal junction and the others regions of interest separately in clinical insight.

Two of the reviewed studies explored the neurophysiological effects of NIBS concurrently to the effect on insight and reported ECT [49] and tDCS [44] effects on the interhemispheric balance using electroencephalography or fMRI and arterial spin labelling. Although the link between NIBS effects on interhemispheric imbalance and insight improvement is not yet reported, these studies provided one mechanistic justification—above probably many others—for the use of NIBS to improve insight. Indeed, previous neuroimaging studies reported that clinical insight can be partly mediated by an interhemispheric imbalance, with an overactive left hemisphere associated with illness denial [11].

Finally, a third mechanistic explanation of NIBS effect on insight could be that NIBS modulates resting-state functional connectivity within the default-mode network (DMN) and particularly reduce DMN increased connectivity associated with poor insight in schizophrenia [72].

### 4.6. Limitations, Potential Biases and Recommendations for Future Studies

Our review has several limitations that should be mentioned. First, given that the review was designed to provide a comprehensive overview of the existing literature on the effects of NIBS on insight in schizophrenia and given the limited data available on this subject, we chose to include case reports and a case series, which limits the generalization of the findings reported here. However, integrating case reports and case series is important to help us understanding the high variability observed across NIBS studies. Second, given the fact that most of the RCTs included in the current review concerned tDCS and that only one RCT has been conducted using another NIBS technique (i.e., rTMS), we were unable to compare the effect of different NIBS techniques with each other.

Our review also suffers from the limitations of the included studies, such as the lack of well-designed RCTs with clinical and/or cognitive insight as primary outcome. In addition, heterogeneity between scales used to measure insight in the studies makes the interpretation of the results all the more complex. Indeed, despite the fact that they nearly all aimed to evaluate the same outcome, i.e., clinical insight, some are unidimensional such as G12 and some are multidimensional such as the VAGUS. They may not all be sensitive enough to capture the multidimensionality of insight. Further studies should consider using standardized and multidimensional scales for the assessment of insight such as the VAGUS insight into psychosis scale [58], with either the 5-item version rated by the clinician (VAGUS-CR: clinician-rated) or the 10-item one rated by the patient (VAGUS-SR: self-reported) (see more information on the scale at the following website: http://vagusonline.com/, accessed on 15 November 2021). This scale has been translated in more than 10 languages, can be completed in less than 5 min and has the potential to detect small and sensitive changes, which are crucial advantages for NIBS studies [58]. Finally, there may be a potential selection bias for NIBS to improve insight in the reviewed studies and more broadly in NIBS trials when recruiting participants. Indeed, participants included in the studies agreed to have NIBS with the expressed purpose of treating the symptoms of schizophrenia (e.g., treatment-refractory auditory hallucinations, persistent negative symptoms or neurocognitive impairment). Thus, they would have had some insight into their illness to recognize that their symptoms were symptoms of illness that could be treated. Such a selection bias is a major obstacle for designing future NIBS trials to improve insight impairment of schizophrenia patients.

Despite these limitations, we believe that the current review provides a comprehensive overview of the effects of NIBS on insight in schizophrenia and could provide a baseline for future works. Major recommendations for further studies conducted in order to assess the effects of NIBS on clinical insight in patients with schizophrenia included the standardization of multidimensional insight assessment along with long-term follow-up assessments. In case of an effect of NIBS on insight, the potential consequences of insight improvement should be carefully evaluated (especially on depressive symptoms, suicidal thoughts and quality of life) and adequately managed using appropriate therapeutic approaches [73].

## 5. Conclusions

NIBS techniques could be considered as emerging tools for enhancing clinical and/or cognitive insight in patients with schizophrenia. This topic needs further research, with well-designed randomized controlled studies including assessments of potential consequences of insight improvement and correlation with symptoms evolution. Given the emerging interest in metacognitive interventions to improve insight, it will be interesting to assess the effect of NIBS as a potentiation of these interventions [74,75].

## Figures and Tables

**Table 1:** Characteristics of the selected studies investigating the effects of NIBS on insight in patients with schizophrenia.

Study	Design	NIBS	Electrode placement * (Size)	Intensity, Freq, Duration	*n* Session	n, Sex M/F, Mean Age	Summary of Results
Rakesh et al., 2013	Case report	tDCS	FP1-F3/T3-P3	2 mA, 20 min	10	1, M, 24	BIS increase after tDCS
Shivakumar et al., 2013	Case report	tDCS	FP1-F3/T3-P3	2 mA, 20 min	10	1, F, 28	BIS increase after tDCS
Bose et al., 2014 ^1^	Open-label	tDCS	FP1-F3/T3-P3 (35 cm^2^)	2 mA, 20 min	10	21, 9/12, 33.1	Significant SAI increase after tDCS (positively correlated with AH improvement).
Agarwal et al., 2016 ^1^	Open-label	tDCS	FP1-F3/T3-P3 (35 cm^2^)	2 mA, 20 min	10	36, 15/21, 33.2	Significant SAI increase after tDCS (positively correlated with AH improvement).
Chang et al., 2018 ^†^	RCT	tDCS	FP1-F3/T3-P3 (35 cm^2^)	2 mA, 20 min	10	A: 30, 14/16, 46.40	Significant SUMD “awareness of disease” and “awareness of positive symptoms” decreases in the active vs sham group after tDCS and at 1 month but not at 3 months.
						S: 30, 13/17, 42.17	
Chang et al., 2019 ^†^	RCT	tDCS	FP1-F3/T3-P3 (35 cm^2^)	2 mA, 20 min	10	A: 30, 14/16, 46.40	Trend towards a significant PANSS-G12 decrease. Significant BCIS-R and R-C index (not BCIS-C) increases after active vs. sham tDCS (almost significant at 1 month).
						S: 30, 13/17, 42.17	
Kao et al., 2020 ^†^	RCT	tDCS	FP1-F3/T3-P3 (35 cm^2^)	2 mA, 20 min	10	A: 30, 14/16, 46.40 S: 30, 13/17, 42.17	Significant PANSS-G12 decrease. Significant SAIQ “presence/outcome” and “need for treatment” (not “worry”) increase after active vs. sham tDCS (reduced to trend level at 1 month).
Kim et al., 2019	RCT	tDCS	F4/F3 or P4/P3 (35 cm^2^)	2 mA, 20 min	1	12, 7/5, 45.0	No significant effect on VAGUS (SR and CR) and BCIS after active vs sham tDCS.
Chang et al., 2021	RCT	tDCS	F3-FP1 + F4-FP2/2 extracephalic (35 cm^2^)	2 mA, 20 min	10	A: 30, 19/11, 44.70	Significant SUMD awareness of the disease, positive symptoms and negative symptoms decreases after active vs. sham tDCS (effects on awareness of the illness and positive symptoms maintained at 1 and 3 months, respectively). No significant effect on SAIQ and BCIS.
						S: 30, 11/19, 45.03	
Sreeraj et al., 2018	Open-label	HD-tDCS	4 * 1 montage: FC3 + FT7 + PO7 + P1/CP5 (ring 3.39 cm^2^)	2 mA, 20 min	10	19, 7/12, 31.79	Significant VAGUS-CR improvement after HD-tDCS.
Haesebaert et al., 2014	Case report	tRNS	FP1-F3/T3-P3 (35 cm^2^)	2 mA, 100–640 Hz, 20 min	10	1, F, 26	SUMD insight into the illness and insight of AH improvement after tRNS and 1 month after.
Kallel et al., 2016	Case series	tACS	F3/F4 (25 cm^2^)	2 mA, 4.5 Hz, 20 min	20	3, 3/0, 24	Mean decrease of 25% in SUMD insight into the illness after tACS.
Dlabac-de Lange et al., 2015	RCT	rTMS	F3 (morning) + F4 (afternoon)	10 Hz, 2000 pulses	30	A: 16, 14/2, 41.8	Significant BIS improvement up to 3 months after active vs. sham rTMS.
						S: 16, 12/4, 32.3	
Gerretsen et al., 2011	Case report	ECT	Bilateral	576 mC (charge), 60 Hz, 6 s	≥18	1, M, 39	Transient awareness of illness, symptoms and medication effects after ECT (SUMD).

* Electrode placement is expressed as the position of the electrode or the coil according to the international EEG electrode placement system. For tDCS, tRNS and tACS, electrode placement is expressed as Anode/Cathode. ^†^ The three articles included the same patient sample. ^1^ The two articles included overlapping patient samples. A: active; AH: auditory hallucinations; BIS: Birchwood Insight Scale; BCIS: Beck Cognitive Insight Scale; BCIS-R: BCIS « self-reflectiveness » subscore; BCIS-C: BCIS « self-certainty » subscore; ECT: electroconvulsivotherapy; F: Female; HD-tDCS: high-definition transcranial Direct Current Stimulation; M: male; *n*: number; NIBS: non-invasive brain stimulation; PANSS-G12: G12 “lack of judgement and insight” item of the Positive and Negative Syndrome Scale; RCT: randomized controlled trial; S: Sham; SAI: Schedule for Assessment of Insight; SAIQ: Self-Appraisal of Illness Questionnaire; SUMD: Scale to Assess Unawareness of Mental Disorder; rTMS: repetitive transcranial magnetic stimulation; tACS: transcranial Alternating Current Stimulation; tDCS: transcranial Direct Current Stimulation; tRNS: transcranial Random-Noise Stimulation; VAGUS-CR: VAGUS insight into psychosis scale—clinician-rated; VAGUS-SR: VAGUS insight into psychosis scale—self-reported.

**Table 2:** Assessment of the methodological quality of included studies with the Kmet’s 14-item QualSyst tool.

Study		1	2	3	4	5	6	7	8	9	10	11	12	13	14	Score
RCT	Dlabac-de Lange et al., 2015	2	2	2	2	2	2	2	2	1	2	2	1	2	2	0.93
	Chang et al., 2018 ^†^	2	2	2	2	1	2	1	2	1	2	2	2	2	2	0.89
	Chang et al., 2019 ^†^	2	2	1	2	1	2	2	2	1	2	2	2	2	2	0.89
	Kim et al., 2019	2	2	2	2	2	2	2	2	0	1	0	1	1	2	0.75
	Kao et al., 2020 ^†^	2	2	1	2	2	2	2	2	1	2	2	2	2	2	0.93
	Chang et al., 2021	2	2	1	2	2	2	2	2	1	2	2	2	2	2	0.93
Non-RCT	Gerretsen et al., 2011	1	0	N/A	1	N/A	N/A	N/A	1	N/A	N/A	N/A	N/A	2	2	0.58
	Rakesh et al., 2013	1	0	N/A	2	N/A	N/A	N/A	2	N/A	N/A	N/A	N/A	2	2	0.75
	Shivakumar et al., 2013	1	0	N/A	2	N/A	N/A	N/A	2	N/A	N/A	N/A	N/A	2	2	0.75
	Bose et al., 2014 ^1^	2	1	1	2	N/A	N/A	N/A	2	1	2	2	N/A	2	2	0.85
	Haesebaert et al., 2014	1	0	N/A	2	N/A	N/A	N/A	2	N/A	N/A	N/A	N/A	2	2	0.75
	Agarwal et al., 2016 ^1^	2	1	1	2	N/A	0	N/A	2	1	2	2	1	2	2	0.75
	Kallel et al., 2016	2	0	N/A	2	N/A	N/A	N/A	2	N/A	N/A	N/A	N/A	2	2	0.83
	Sreeraj et al., 2018	1	1	1	2	N/A	N/A	N/A	2	1	2	2	N/A	2	2	0.80

The 14 evaluation criteria are as follows: 1. Objective sufficiently described? 2. Study design evident and appropriate? 3. Method of subject/comparison group selection described and appropriate? 4. Subject (and comparison group, if applicable) characteristics sufficiently described? 5. If interventional random allocation was possible, was it described? 6. If interventional blinding of investigators was possible, was it reported? 7. If interventional blinding of subjects was possible, was it reported? 8. Outcome and (if applicable) exposure measure(s) well defined and robust to measurement/misclassification bias? Means of assessment reported? 9. Sample size appropriate? 10. Analytic methods described/justified and appropriate? 11. Some estimate of variance is reported for the main results? 12. Controlled for confounding? 13. Results reported in sufficient detail? 14. Conclusions supported by the results? Criteria are rated as 0 = no, 1 = partial, 2 = yes and N/A = Not applicable. ^†^ The three articles included the same patient sample; ^1^ the two articles included overlapping patient samples.

## Data Availability

All the data extracted from the included studies are provided in the manuscript (text and table).
